# Valuable natural products from marine and freshwater macroalgae obtained from supercritical fluid extracts

**DOI:** 10.1007/s10811-017-1257-5

**Published:** 2017-09-07

**Authors:** Beata Messyasz, Izabela Michalak, Bogusława Łęska, Grzegorz Schroeder, Bogusława Górka, Karolina Korzeniowska, Jacek Lipok, Piotr Wieczorek, Edward Rój, Radosław Wilk, Agnieszka Dobrzyńska-Inger, Henryk Górecki, Katarzyna Chojnacka

**Affiliations:** 1 Faculty of Biology, Department of Hydrobiology, Adam Mickiewicz University in Poznan, Umultowska 89, 61-614 Poznan, Poland; 2 Institute of Inorganic Technology and Mineral Fertilizers, Wrocław University of Technology, Smoluchowskiego 25, 50-372 Wrocław, Poland; 3 Faculty of Chemistry, Adam Mickiewicz University in Poznan, Umultowska 89b, 61-614 Poznan, Poland; 4 Faculty of Chemistry, Opole University, Oleska 48, 45-051 Opole, Poland; 5 Supercritical Extraction Department, Fertilizer Research Institute, Aleja Tysiaclecia Panstwa Polskiego 13a, 24-110 Pulawy, Poland

**Keywords:** Antioxidant properties, Bioactive compounds, DPPH, Fatty acids, Polyphenols, Supercritical fluid extraction

## Abstract

The biologically active compounds (fatty acids, pigments, phenolics, and flavonoid content) were studied in supercritical fluid extracts from the biomass of marine (*Ulva clathrata*, *Cladophora glomerata*, *Polysiphonia fucoides*, and their multi-species mixture) and freshwater (*C. glomerata*) macroalgae. Different extraction techniques were used in order to compare differences in the biologically active compound composition of the macroalgal extracts. The results indicated that the saturated and unsaturated fatty acids ranged from C9:0 to C22:0. The analysis of differences in the composition of unsaturated to saturated fatty acids in extracts showed that palmitic acid (C16:0) and oleic acid (C18:1, n-9) reached the highest value not only in marine monospecies and multi-species biomass but also in the freshwater macroalga *C. glomerata*. When comparing the similarity between the concentration of fatty acids and the ratio of the concentration of unsaturated fatty acids to saturated in macroalgal extracts, we found small but not statistically significant variations in values between years (up to 10%). This is acceptable for applications as a stable raw material for industrial purposes. Significantly higher values of fatty acids, carotenoids, and chlorophylls were obtained in the case of SC-CO_2_ extraction. The active ingredients of polyphenols, possessing antioxidant activity ranged from approximately 2–4%. Moreover, flavonoids represented less than 10% of the total content of polyphenolic compounds. The extraction efficiency of polyphenols was higher from a mixture of marine algae for the ultrasound-assisted extraction compared to freshwater. All these findings show that marine and freshwater macroalgae, as a raw material, have the optimal biologically active compounds composition for cosmetics.

## Introduction

According to literature, fatty acids and pigments are extracted mainly from the biomass of microalgae. Little attention in this respect has been paid to macroalgae. A comparison of the interest in micro- and macroalgae is presented in Table 1. In some cases, instead of the word “macroalgae” the word “seaweed” is used. In the last 15 years, the words “extraction of fatty acids/from macroalgae” appeared in the topic scientific papers 26 times, “extraction of fatty acids/from seaweed” 47 times, whereas “extraction of fatty acids/from microalgae” 435 times (Source: Web of Knowledge, 11 April 2017).

**Table 1:** Number of papers on extraction of valuable natural products from algae

Extraction of/from	Number of papers on^a^		
	Macroalgae	Seaweed	Microalgae
Fatty acids	26	47	435
Pigments	17	31	106
Carotenoids	13	38	138
Chlorophyll	9	17	94
Polyphenols	13	43	9
Flavonoids	4	18	2

^a^According to the Web of Knowledge, 11 April 2017

However, the biomass of macroalgae is also a rich source of polyunsaturated fatty acids—PUFAs (both: omega-3 fatty acids: eicosapentaenoic acid (EPA) and docosahexaenoic acid (DHA) and omega-6 fatty acid: γ-linolenic acid (GLA) and arachidonic acid (AA) (Pereira et al. 2012). For example, the fatty acid profile of green seaweed *Cladophora rupestris* (L.) Kützing lipidic extract including palmitic, myristic, oleic, α-linolenic, palmitoleic, and linoleic acids (Stabili et al. 2014). Horincar et al. (2014) have reported that the *Ulva intestinalis* L. extract had a greater content of mono- and polyunsaturated fatty acids of around 46.0%, as compared with 42.0% for *Cladophora vagabunda* (L.) Hoek and 31.9% for *Ceramium virgatum* Hooker & Harvey. The most abundant fatty acids were palmitic acid (C16:0), arachidonic acid (C20:4n-6), and oleic acid (C18:1ω-9*cis*). Chemical characterization of other lipidic extract of *Gracilariopsis longissima* (Gmelin) Steentoft, Irvine, & Farnham has revealed that palmitic acid methyl ester (16:0) was the predominant saturated fatty acid (42%), while, from among monounsaturated fatty acids, oleic acid methyl ester (18:1) prevailed (8.5%) (Stabili et al. 2012). In this paper, special attention was paid to the biomass of macroalgae collected from the Baltic Sea (Poland, southern Baltic), belonging to the taxa *Polysiphonia*, *Ulva*, and *Cladophora* and the freshwater macroalga, *Cladohora*, from Lake Oporzynskie (West Poland). This biomass could constitute a valuable source of biologically active compounds (especially fatty acids and pigments: carotenoids and chlorophyll) which could be potentially used by the food, pharmaceutical, and cosmetic industries.

It should be noted that different extraction techniques can be used in order to obtain biologically active compounds from the biomass of algae. The choice of the appropriate method should depend on the nature of the extracted compound (Kadam et al. 2013; Ibañez et al. 2012). Until now, biologically active compounds have been extracted from the biomass of algae mainly by conventional solvent extraction (with the use of organic solvents: i.e., petroleum ether, hexane, cyclohexane, isooctane, toluene, benzene, diethyl ether, dichloromethane, isopropanol, chloroform, acetone, methanol, ethanol) (Stabili et al. 2012, 2014; Horincar et al. 2014). The second method is hydrolysis carried out under alkaline, neutral, or acidic conditions (Booth 1969). However, according to current trends, the use of organic solvent should be minimized. The solution could be the application of supercritical fluid extraction (SFE) with CO_2_ as a green solvent. It has been shown that SFE is a suitable technology for extraction of nutraceuticals. Bioactive compounds can also be extracted without any loss of volatility and their degradation. SFE offers a high extraction rate and high yield and is an eco-friendly technology with minimal or no use of organic solvents (Kadam et al. 2013). Some examples of the use of SFE for the extraction of lipids from the biomass of macroalgae are summarized in Table 2.

**Table 2:** SFE extraction of biomass of macroalgae—review of literature reports

Extraction	Macroalgae	Temp.	Pressure	Extraction time	Extracted compound	Reference
SFE with CO_2_	*Sargassum hemiphyllum*	40–50 °C	24.1–37.9 MPa	60 min	Fatty acid profiles of lipids	Cheung et al. 1998
SFE with CO_2_	*Hypnea charoides*	40–50 °C	24.1–37.9 MPa	120 min	Lipids	Cheung 1999
SFE with CO_2_	*Dictyopteris membranacea*	40 °C	9.1 and 10.4 MPa	30 min	Volatile metabolites (oils)	El Hattab et al. 2007
SFE with CO_2_	*Plocamium cartilagineum*	40 °C	40.53 MPa	180 min	Halogenated monoterpenes	Gao and Okuda 2001
SFE with CO_2_	*Chaetomorpha linum*	50 °C	2.60 MPa	420 min	Oil	Aresta et al. 2005
SFE with CO_2_	*Bangiaatro purpurea* *Porphyra angusta* *P. dentate* *Helminthocladia australis* *Liagora orientalis* *L. boergesenii* *Scinaia monoliformis* *Galaxaura cylindrica* *Grateloupia filicina* *Halymenia ceylanica*	55 °C	3.45 MPa	180 min	Fatty acid	Chen and Chou 2002
SFE with CO_2_	*Polysiphonia nigrescens* *Ulva clathrata* *Cladophora* sp.	40 °C	50.00 MPa	300, 360, and 810 min	Auxins cytokinins polyphenols micro- and macroelements	Michalak et al. 2016
SFE with CO_2_	*Sargassum muticum*	55 °C	40.00 MPa	120 min	Fucoxanthin	Pérez-López et al. 2014; Esquivel-Hernández et al. 2017
SFE with CO_2_	*Cladophora glomerata* *Ulva flexuosa* *Chara fragilis*	40 °C	30.00 MPa	120 min	Fucoxanthin and astacene antioxidant and deinoxanthin, siphonaxanthin phenols and carotenoids	Fabrowska et al. 2016

In relation to biological activity, mainly the antioxidant properties, an important group are phenolic compounds and flavonoids. Polymers of phenolic compounds, polyphenols, are a large and diverse group of secondary metabolites produced by plants and fungi, containing in their structure at least one hydroxyl group bonded directly to the aromatic ring. Flavonoids are related to the class of polyphenolic compounds, but their chemical structure consists mainly of two phenyl rings and a heterocyclic ring, which may be substituted at different positions mainly with hydroxyl and methyl groups (Kumar and Pandey 2013).

Among the variety of biological properties of polyphenols, they have a strong radical scavenging ability; therefore, they exhibit noteworthy antioxidant activity. It may occur due to reducing ability, binding of free radicals, chelation of metal ions, stabilization of the free radicals, and inhibition of oxidases. For the above reason, this group of compounds takes part in prevention of many diseases, mostly related to oxidative stress like those caused by harmful solar radiation or attack by pathogens (Lobo et al. 2010).

Colorimetric methods are very useful for identification of classes of compounds, e.g., polyphenols, flavonoids, chlorophylls, or for the evaluation of antioxidant properties of particular extracts or solutions. There are many methods, which allow measurements of the antioxidant activity of single substances and mixtures of compounds. The total quantity of phenolic compounds was determined by using the Folin-Ciocalteu test, using the ferric reducing ability of plasma (FRAP) protocol, 2,2′-azino-bis (3-ethylbenzothiazoline-6-sulphonic acid) diammonium salt (ABTS) protocol, or 2,2-diphenyl-1-picrylohydrazyl (DPPH) test (Chakraborty et al. 2015). Some of these analyses were conducted in our research, in order to check the radical scavenging ability of the extracts studied and establish the content of biologically active components in macroalgae extracts.

Nonetheless, the results of measurements performed with the use of the above mentioned methods might be compared, when referred to the same, well-defined reference material. The most commonly used reference substance is Trolox (6-hydroxy-2,5,7,8-tetramethylchroman-2-carboxylic acid), a synthetic derivative of tocopherol. The antioxidant content is expressed as the amount of Trolox equivalents—TEAC per weight or volume of the sample. Sometimes ascorbic acid, which is considered as powerful antioxidant, is also used for comparison. It is difficult to indicate the best method, which is charged with the least possible error, since any factor may affect the results. The light and oxygen access, pH, and type of solvent are the most common considered ones (Cybul and Nowak 2008).

The aim of this study was to extract by supercritical fluid extraction with CO_2_ biomass of marine (from the Baltic Sea) and freshwater macroalgae in order to isolate biologically active compounds that have potential applications as natural components of cosmetics and pharmaceuticals. In our previous work, the same extract was examined as a natural plant biostimulant. The utilitarian properties were checked in germination tests with garden cress (*Lepidium sativum* L.) and wheat (*Triticum aestivum* L.). The contents of inorganic (macro- and microelements) and organic (plant hormones: auxins and cytokinins; polyphenols) compounds were determined (Michalak et al. 2016).

## Materials and methods

### Collection of algae and extraction

Collection of individual species (manually) and the mixture of algae (industrial, mechanical collection of biomass) from the Baltic Sea (*Ulva clathrata* (Roth) Ag., *Cladophora glomerata* (L.) Kütz., *Polysiphonia fucoides* (Hudson) Greville) and the production of extract by classical and supercritical fluid extraction, which involved the pretreatment of biomass was described previously (Michalak et al. 2016). Freshwater *C*. *glomerata* thalli were collected from the shallow Lake Oporzynskie (N 52° 55′ 70″, E 17° 09′ 60″) situated in the northern part of the Wielkopolska region (western Poland) in the July–August period of 2013 and 2014 when algal biomass was at its annual maximum. Characterization of physical and chemical parameters during the intensive development of *C. glomerata* in the lake was described earlier (Messyasz et al. 2015a; Pikosz and Messyasz 2016). The algae samples collected from the water were put into plastic container with water coming from the same habitat in the ratio of 3:1 and transported to the laboratory. Next, the thalli were repeatedly rinsed with distilled water to separate any abiotic particles attached to them. The washed fresh algal biomass was weighed immediately and a small portion (5 g) was used for microscopic analysis, using a light microscope (Zeiss Axioskop 2 MOT), at ×200 and ×400 magnification and checking their surface for the presence of microscopic algae. To identify the alga species, the length and width of the cells were measured and algal samples were stained with Lugol’s solution to determine the number of pyrenoids, or with acetocarmine to determine the number of nuclei. Next, the extant material was dried in an oven until water content of biomass was lower than 15% (*w*/*w*).

In conventional extraction, macroalgae were extracted with 200 mL of ethanol as a solvent for 3 h.

### Supercritical fluid extraction of Baltic algae

In the Fertilizer Research Institute in Puławy, Baltic macroalgal biomass was processed as already described by Michalak et al. (2016). After optimization of the method, the best process parameters were chosen. In this study, seaweed extract obtained in supercritical fluid extraction (in which fine-grained grist was used) was examined. The summary of the SFE of Baltic seaweed extraction is as follows: pressure 500 bar, temperature 40 °C, mass of post extraction remains 9.695 kg, extract mass 179 g, extraction capacity 1.76%, total capacity with total mass loss 4.78%; solvent: CO_2_ and ethanol.

### Ultrasound-assisted extraction (UAE)

Ultrasound-assisted extracts were made out of raw, powdered material of Baltic seaweed and freshwater *C*. *glomerata*. For the extract preparation for colorimetric analysis, 10 g of dry weight of material was extracted in the ultrasonic bath (Cole Parmer, 8891, USA) with two portions of methanol as a solvent (2 × 100 mL), over a total time of 1 h. After 30 min, the first part of solvent was removed and new portion was added to continue the extraction for another 30 min. The temperature of the ultrasonic bath did not exceed 35 °C. The extracts were filtrated and the filtrates were combined. The solvent was removed on the rotary evaporator. In order to prepare samples for colorimetric analysis, the methanolic solutions of extracts were made up to a concentration of 10.00 ± 0.06 mg mL^−1^.

### Analytical methods

#### Determination of fatty acids in extracts from Baltic seaweed

Sigma-Aldrich reagents and standards were used in the fatty acid analysis. Extract − 5 mL was evaporated under reduced pressure (< 1 mmHg) at a temperature of < 40 °C until constant weight. To the dry residue, 0.5 mL of *tert*-butyl methyl ether (MTBE) was added. Next, 0.25 mL of 0.2 M solution of trimethylsulfonium hydroxide in methanol as a derivatizing agent was added. After 5 min of stirring at room temperature, a solution of 10 μL of methyl undecanoate in MTBE with concentration of methyl undecanoate 42.6 mg mL^−1^ MTBE was added. The methyl undecanoate as the internal standard was used. Fatty acids as methyl esters were determined using a Varian gas chromatograph GC 450. Operating parameters of the chromatograph are as follows: injector temperature, 250 °C; split, 1:50; carrier gas, He, flow rate 1 mL min^−1^; column, Varian VF-WAXms, 30 m × 0.53 mm, film thickness 1 μm; temperature program, 50 °C isothermal for 2 min; linear gradient of 10 °C min^−1^ to 250 °C (20 min), isothermal 250 °C for 23 min; detector, FID detector temperature 250 °C; injection volume of 2 μL of sample. The identification of methyl esters of fatty acids was performed according to retention times of standards. The following acid standards (as methyl esters) were used: butyric acid (C4:0), valeric acid (C5:0), caproic (C6:0), caprylic acid (C8:0), nonanoic (C9:0), capric (C10:0), undecanoic (C11:0), lauric (C12:0), myristic (C14:0), palmitic (C16:0), margarine (C17:0), stearic (C18:0), arachidic (C20:0), behenic (C22:0), myristoleic (C14:1, n-5), palmitoleic (C16:1, n-7), oleic (C18:1, n-9), vaccenic (C18:1, n-7) petroselinic (C18:1, n-12) *cis*-11-eicosenoic (C20:1, n-9), berucic acid (C22:1, n-9), nervonic (C24:1, n-9), linoleic (C18:2, n-6), α-linolenic (C18:3 n-3), γ-linolenic acid (C18:3, n-6), stearidonic (C18:4, n-3), *cis cis*-11,14-eicosadienoic (C20:2, n-6), arachidonic (C20:4, n-6); all-*cis*-5,8,11,14,17-eicosapentaenoic (C20:5, n-3), all-*cis*-7,10,13,16,19-docosapentaenoic (C22:5, n-3), all-*cis*-4,7,10,13,16,19-docosahexaenoic acid (C22:6, n-3).

#### Determination of carotenoids and chlorophyll a and b in extracts

The determination of the total concentration of carotenoids and chlorophyll a was conducted according to the Wellburn method (Wellburn 1994) as described by Macias-Sanchez et al. (2007, 2008) by measuring the absorbance of the different samples using a Cary Spectrophotometer (wavelength from 330 to 800 nm).

#### Determination of total phenolic compounds (TPC) in extracts

Determination of total polyphenolic compounds in the extracts from Baltic seaweeds and freshwater *C. glomerata* were obtained using SFE-CO_2_ extraction. UAE extraction was performed using the method described in (Sim et al. 2010) with slight modifications. For these measurements, Baltic seaweed SFE-CO_2_ extract obtained in 2014 was used, for which phenolic compounds content was determined (Michalak et al. 2016).

The calibration curve was prepared by dissolving gallic acid in 70% methanol to obtain the stock solution with the concentration of 1 mg mL^−1^ After that, the subsequent dilutions were made in the range of concentrations from 0.1 to 1 mg mL^−1^. To the reaction vessel was added 20 μL of gallic acid solution with a particular concentration, 1.58 mL of distilled water, 0.1 mL of Folin-Ciocalteu reagent (Folin-Ciocalteu Phenol Reagent, POCH, Poland) and 0.3 mL of saturated solution of sodium bicarbonate (Na_2_CO_3_, POCH, Poland). The final reaction volume was 2 mL and the final concentration of gallic acid ranged between 0.001 and 0.01 mg mL^−1^. The reaction mixture with real samples were prepared as the samples for the calibration curve (*y* = 100.28*x* + 0.0412; *R* ^2^ = 0.9975), the 20 μL of 10.00 mg mL^−1^ extract solution was added instead of the gallic acid solution. The result was expressed as gallic acid equivalent (GAE) using the following equation: *C* [mg GAE ${g}_{{\mathrm{extract}}^{-1}}$] = *C*_i_ [mg mL^−1^] * (*V*_1_ [mL]/*V*_2_ [mL]) * (*V*_3_ [mL]/*m* [g]), where *C*_*i*_ [mg mL^−1^] is the concentration from calibration curve, *V*_1_ is the total volume of reaction vessel, *V*_2_ is the volume of extract/standard added to the reaction, *V*_3_ is the volume in which the extract was dissolved, and *m* is the mass of the extract dissolved in *V*_3_ in order to prepare real sample of extract. After keeping the reaction vessels in the darkness for 2 h in the room temperature, the samples were measured with UV/VIS spectrometer at 760 nm. Each sample was prepared and measured in triplicate. Data are mean ± SD values.

#### Determination of total flavonoids (FC) in extracts

The aluminum chloride method was used for the determination of the total flavonoid content of the sample extracts. This method is based on the formation of a complex between the aluminum ion, Al^3+^, and the carbonyl and hydroxyl groups of flavones and flavonols, which in a results in an yellow color of the solution. Total content of these compounds was determined by the protocol of Baba and Shahid (2015) with some modifications. The volume of the reaction mixture was 2 mL and consisted of 0.8 mL of methanol, 0.2 mL of 10.00 mg mL^−1^ extract or standard solution, and 0.06 mL of 5% NaNO_2_. The reaction vessel was placed in the dark for 5 min. After that time, 0.06 mL of 10% AlCl_3_ was added and again left in the dark for 5 min. In the next step, 0.4 mL of 1 M NaOH and 0.28 mL of methanol were added and the mixture was placed in a dark place for another 15 min. All of the samples were filtered and measured against a blank sample (methanol instead of extract or standard). The calibration curve was prepared using quercetin as a standard in the range of concentrations 0.05–0.25 mg mL^−1^ (*y* = 3.204*x* + 0.0024; *R* ^2^ = 0.9975) and the result was expressed as quercetin equivalent (QE) using the following equation: *C* [mg QE ${g}_{{\mathrm{extract}}^{-1}}$] = *C*_i_ [mg mL^−1^] * (*V*_1_ [mL]/*V*_2_ [mL]) * (*V*_3_ [mL]/*m* [g]). Each sample was prepared and measured in triplicate. Data are mean ± SD values.

#### Determination of antioxidant activity of extracts

One of the most common methods used to evaluate the antioxidant activity is a test with 2,2-diphenyl-1-picrylhydrazyl (DPPH), which was used for preliminary evaluation of the potential of the antioxidant extracts of algae. The solution of free radicals and DPPH has a purple color. During the reduction reaction with the substance with antioxidant properties, component of tested extract, it changes the color to yellow and is spectrophotometrically measured. The reaction mixture was prepared by adding 200 μL of sample (10.00 mg mL^−1^ extract), 2 mL of methanolic solution of DPPH (0.04 mg mL^−1^; DPPH, Sigma, Poland) and left for 30 min in the dark. The measurements were conducted at the wavelength of 517 nm.

In addition, the antioxidant content in the analyzed samples is expressed as Trolox (6-hydroxy-2,5,7,8-tetramethylchroman-2-carboxylic acid) equivalents (TEAC) per unit volume [mg TEAC (100 mL)^−1^ of extract], determining the concentration from the standard curve for Trolox. The calibration curve equation was *y* = −0.0774*x* + 0.8682 with the regression coefficient 0.9966. The antioxidant properties of ascorbic acid, which is a potent antioxidant were used for the comparison. All of the samples were measured in triplicate. Data are mean ± SD values.

## Results

### Fatty acid composition in marine and freshwater algae extracts

The differences in fatty acid composition were observed depending on the extraction method and extracted algal biomass (monospecies, multi-species). In our studies, the saturated fatty acids ranged from C9:0 to C22:0. The highest concentration of saturated fatty acids (mainly C16:0) were observed for individual marine species *U. clathrata*, *C. glomerata*, and also *P. fucoides* (Table 3). Analogous results were also obtained for Baltic multi-species biomass extraction, using supercritical CO_2_. All extracts contained high concentrations of unsaturated fatty acids, especially C16:1 (n-7) and C18:1 (n-3). C16:0 and C18:1 (n-9) fatty acids were detected in the largest percentage in the case of supercritical fluid extraction, and the C18:0, C18:4 (n-3), and C22:6 (n-3) fatty acids were found in the lowest amount in solvent extraction. Traditional extraction of marine multi-species biomass by ethanol as compared to that with using SC-CO_2_ was characterized by a substantially lower yield of fatty acids.

**Table 3:** Fatty acid composition in extract of algae biomass collection of the Baltic seaweed in years 2013 and 2014. Values presented are mean ± standard deviation, *n* = 3

% weight of fatty acids in dry matter of the extract										
Component	The biomass of one species						Biomass of multi-species			
	*Ulva clathrata*		*Cladophora glomerata*		*Polysiphonia fucoides*		Industrial (mechanical) collection of biomass			
Collection dates/extractions method	2013 ES	2014 ES	2013 ES	2014 ES	2013 ES	2014 ES	2013 ES	2014 ES	2013 SC-CO_2_	2014 SC-CO_2_
C9:0	5.9 ± 0.56	6.5 ± 0.4	2.7 ± 0.3	3.0 ± 0.32	1.7 ± 0.2	1.8 ± 0.2	0.2 ± 0.04	0.2 ± 0.05	2.2 ± 0.3	2.8 ± 0.4
C10:0	<LLD	<LLD	<LLD	<LLD	<LLD	<LLD	<LLD	0.1 ± 0.01	1.1 ± 0.3	1.5 ± 0. 5
C11:0	<LLD	<LLD	<LLD	<LLD	<LLD	<LLD	<LLD	<LLD	<LLD	< 0.1
C12:0	<LLD	<LLD	<LLD	<LLD	<LLD	<LLD	< 0.1	0.1 ± 0.01	1.5 ± 0.23	0.9 ± 0.19
C14:0	3.2 ± 0.5	3.3 ± 0.6	2.0 ± 0.4	2.7 ± 0.4	1.0 ± 0.2	1.0 ± 0.2	0.5 ± 0.1	0.6 ± 0.1	6.7 ± 1.210	5.9 ± 1.11
C16:0	22.7 ± 1.8	21.8 ± 1.7	6.0 ± 0.9	8.2 ± 1.2	5.8 ± 0.6	5.8 ± 0.6	2.6 ± 0.2	2.6 ± 0.2	27.8 ± 2.0	33.1 ± 2.3
C18:0	0.9 ± 0.1	0.9 ± 0.1	0.1 ± 0.08	0.1 ± 0.01	0.1 ± 0.01	0.1 ± 0.1	0.4 ± 0.1	0.4 ± 0.1	6.1 ± 1.2	6.2 ± 1.2
C20:0	<LLD	<LLD	<LLD	<LLD	<LLD	<LLD	< 0.1	0.1 ± 0.1	0.2 ± 0.1	0.2 ± 0.07
C22:0	<LLD	<LLD	<LLD	<LLD	<LLD	<LLD	0.1 ± 0.03	0.1 ± 0.05	0.4 ± 0.1	0.1 ± 0.1
C16:1 (n-7)	22.2 ± 2.3	25.8 ± 2.4	2.0 ± 0.3	2.0 ± 0.3	1.9 ± 0.3	2.0 ± 0.3	0.9 ± 0.1	0.9 ± 0.1	7.4 ± 1.2	5.4 ± 1.1
C18:1 (n-9)	4.5 ± 0.4	3.8 ± 0.4	1.3 ± 0.2	1.0 ± 0.09	1.3 ± 0.2	1.3 ± 0.2	0.7 ± 0.1	0.5 ± 0.1	20.3 ± 2.2	21.8 ± 2.2
C18:2 (n-6)	2.4 ± 0.3	2.4 ± 0.3	1.9 ± 0.1	1.5 ± 0.2	1.6 ± 0.2	1.7 ± 0.2	0.4 ± 0.1	0.4 ± 0.1	5.8 ± 0.5	4.8 ± 0.5
C18:3 (n-3)	1.9 ± 0.2	2.0 ± 0.2	4.0 ± 0.3	4.0 ± 0.3	3.0 ± 0.2	2.9 ± 0.2	0.2 ± 0.05	0.1 ± 0.04	1.3 ± 0.1	1.1 ± 0.1
C18:3 (n-6)	<LLD	<LLD	<LLD	<LLD	<LLD	<LLD	<LLD	<LLD	<LLD	<LLD
C18:4 (n-3)	0.5 ± 0.1	0.9 ± 0.1	1.2 ± 0.08	1.6 ± 0.1	1.2 ± 0.1	1.5 ± 0.1	0.3 ± 0.07	0.2 ± 0.05	<LLD	0.2 ± 0.04
C20:4 (n-6)	<LLD	<LLD	<LLD	<LLD	<LLD	<LLD	<LLD	<LLD	0.5 ± 0.1	0.2 ± 0.1
C20:5 (n-3)	<LLD	<LLD	<LLD	<LLD	<LLD	< 0.1	<LLD	<LLD	<LLD	<LLD
C22:1 (n-9)	<LLD	<LLD	<LLD	<LLD	<LLD	< 0.1	<LLD	<LLD	0.3 ± 0.08	0.3 ± 0.07
C22:6 (n-3)	<LLD	<LLD	<LLD	<LLD	0.1 ± 0.01	0.1 ± 0.01	<LLD	<LLD	<LLD	<LLD
The content of fatty acid (% weight) in the dry matter of the extract	64.2 ± 6.1	67.4 ± 6.3	21.2 ± 2.1	24.1 ± 2.1	17.7 ± 2.0	18.2 ± 1.9	6.3 ± 0.7	5.7 ± 0.6	81.6 ± 7.9	84.50 ± 8.0
PIN/SAT^a^	0.96	1.07	0.96	0.84	1.05	1.09	0.65	0.58	0.77	0.66

*<LLD* below lower limit of detection (limit of detection 0.1% weight, error in determining analytes = 0.1% weight); *ES* extraction Soxhlet method, solvent EtOH; *SC*-*CO*_*2*_ supercritical fluid extraction with CO_2_

^a^[PIN/SAT] Polyunsaturated/Saturated fatty acid ratio in the dry matter of the extract

**Fig. 1:**
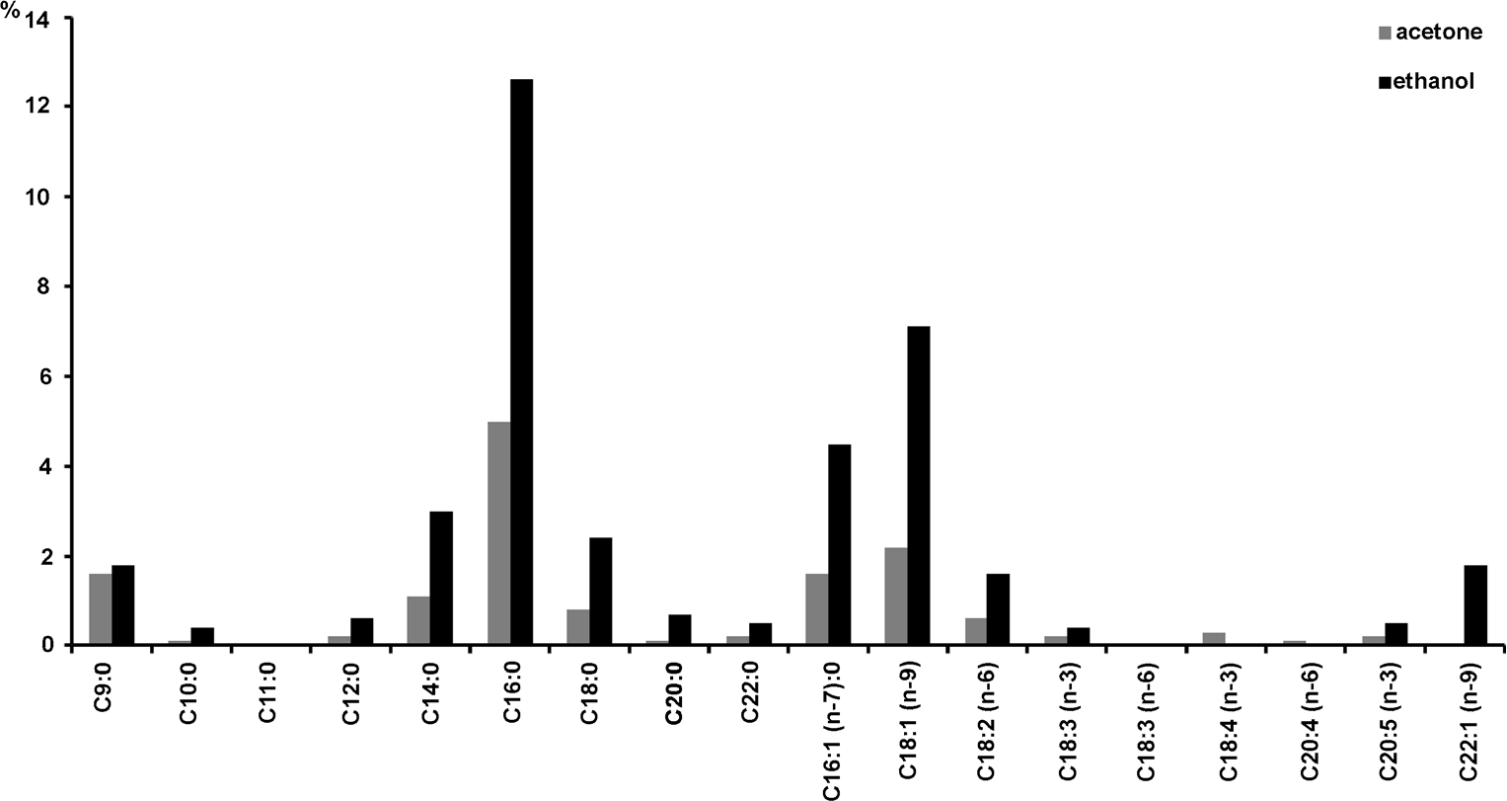
Fatty acid composition in extract (extraction Soxhlet method: solvent acetone, ethanol) of multi-species marine macroalgae biomass (% weight of fatty acids in dry matter of the extract)

**Fig. 2:**
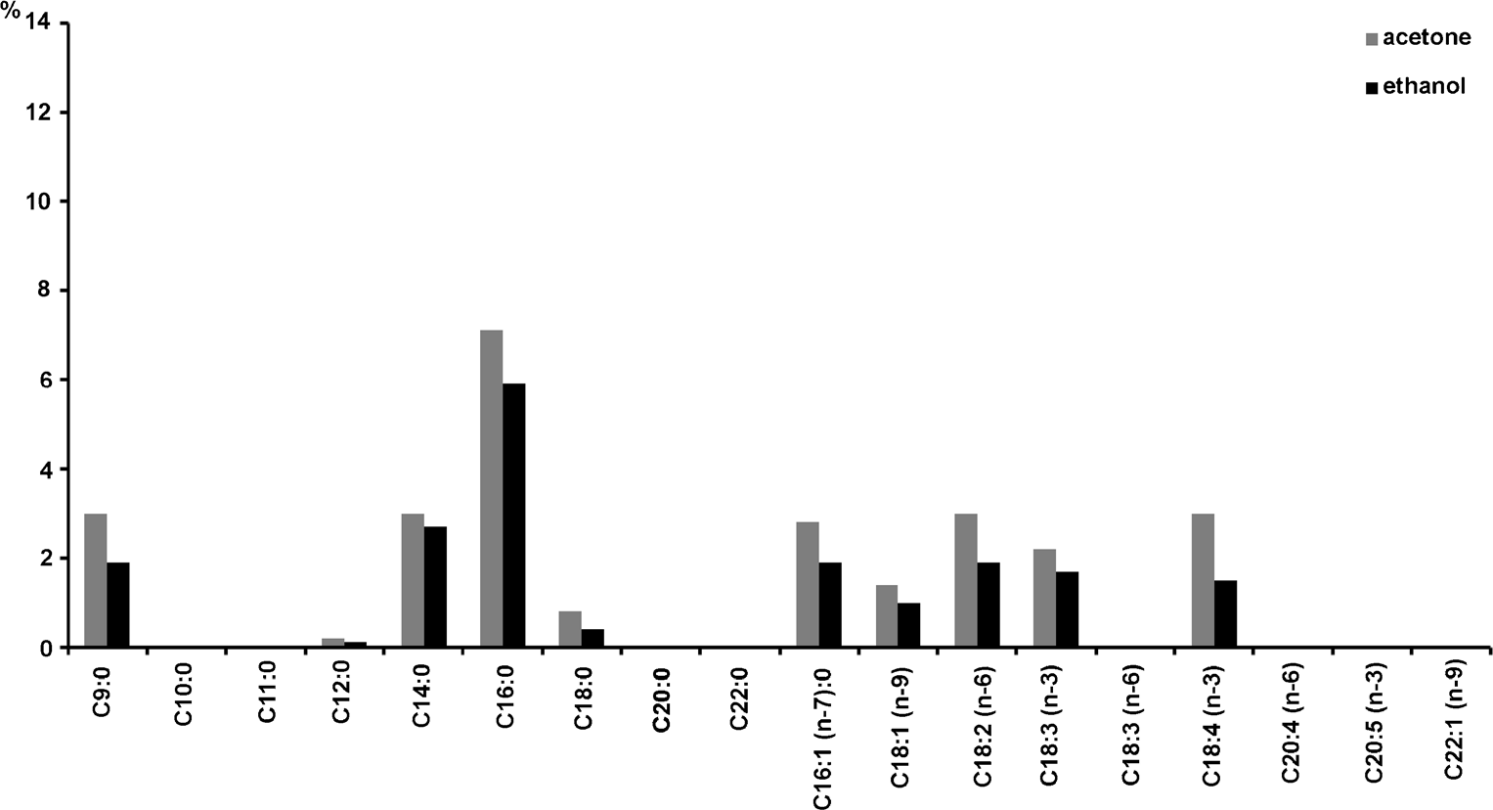
Fatty acid composition in extract (extraction Soxhlet method: solvent acetone, ethanol) of marine *Ulva clathrata* biomass (% weight of fatty acids in dry matter of the extract)

In order to compare the fatty acid compositions in marine biomass of multi-species macroalgae and single algal species *U*. *clathrata*, the samples of the two types of biomass were subjected to the Soxhlet extraction method with the use of two different solvents (acetone, ethanol). Higher values of unsaturated/saturated fatty acid ratio were obtained for the dry matter of the extract from biomass of marine algae (1.75 ± 0.36) and biomass of *U. clathrata* (1.75 ± 0.35), using ethanol than when using acetone as a solvent (1.38 ± 0.23 and 1.13 ± 0.17, respectively). The fatty acids C16:0, C18:0, C16:1 (n-7), C18:1 (n-9), and C22:1 (n-9) occurred in the highest amounts among other unsaturated fatty acids in the extracts obtained from multi-species marine biomass (Fig. 1). On the other hand, fatty acids, which were obtained in low amounts, belonged to C20:0 and C22:0. Additionally, in the *U. clathrata* biomass, the percentage weight of fatty acids in dry matter of the extract was often smaller than that in the mixture of marine macroalgae. The extraction efficiency of C18:3 and C18:4 fatty acids was a little better with acetone as a solvent when applying the Soxhlet method (Fig. 2).

**Fig. 3:**
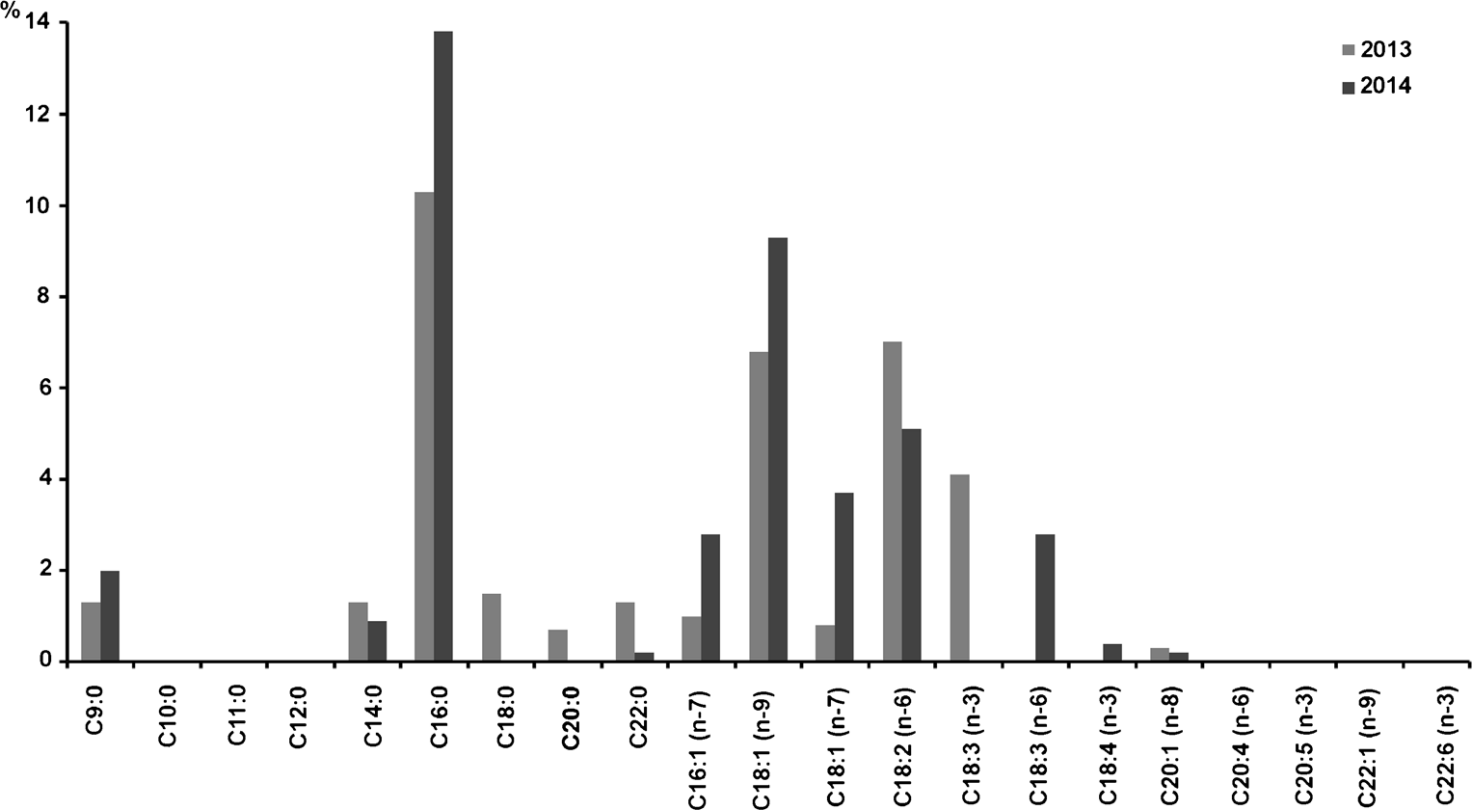
Fatty acid composition in SC-CO_2_ extract of freshwater *Cladophora* sp. biomass in years 2013 and 2014 (% weight of fatty acids in dry matter of the extract)

The concentration of fatty acid and the ratio of the concentration of unsaturated fatty acids to saturated ones in the extracts obtained by various methods, for the biomass samples of one species of freshwater alga *C. glomerata* (Table 4, Fig. 3) collected in the same way (the time from harvest to dry biomass approx. 4 h) in 2013 and 2014 differed approximately by 10%. In the freshwater *C. glomerata*, the fatty acid composition was dominated by the highest amounts of C16:0 (high values also in marine mono- and multi-species biomass), and subsequently C18:1 (n-9) (high values also in marine mono- and multi-species biomass), C18:1 (n-7), C18:2 (n-6), C18:3 (n-3), and C18:3 (n-6) (Fig. 3).

**Table 4:** The content of fatty acid (% weight in the dry matter of the extract) and the polyunsaturated/saturated fatty acid ratio in the dry matter of the extract of freshwater *Cladophora glomerata* biomass extract in years 2013 and 2014. Values presented are mean ± standard deviation, *n* = 3

Extraction method	Fatty acids (%)		Polyunsaturated/saturated fatty acid ratio	
	2013	2014	2013	2014
Soxhlet, hexane	28.8 ± 0.63	24.5 ± 0.78	1.24 ± 0.22	1.57 ± 0.02
Soxhlet, acetone	34.0 ± 1.02	27.4 ± 0.91	1.88 ± 0.12	1.52 ± 0.01
Soxhlet, ethanol	21.2 ± 0.71	24.1 ± 0.87	0.96 ± 0.01	0.84 ± 0.01
SC-CO_2_	36.4 ± 1.32	41.2 ± 2.24	1.21 ± 0.11	1.42 ± 0.02

### Pigments in extracts from Baltic seaweed

The extraction of carotenoids and chlorophylls using carbon dioxide is an alternative to traditional Soxhlet extraction. The yields of the carotenoid and chlorophyll extractions obtained from the three macroalgae and biomass of multi-species from Baltic seaweeds are shown in Table 5. For Soxhlet extraction by ethanol, the highest concentration of chlorophyll was observed for *C. glomerata*, while for carotenoids for *P. fucoides.* The biomass of multi-species seaweeds from Baltic Sea obtained by SC-CO_2_ extraction contained the highest amounts of carotenoids and chlorophylls extraction relative to the extracts obtained by the Soxhlet method with ethanol.

**Table 5:** The contents of chlorophyll and carotenoids in extract of the dry marine algal biomass (mg g^−1^). Values presented are mean ± standard deviation, *n* = 3

Component	The biomass of one species			Biomass of multi-species	
	*Ulva clathrata*	*Cladophora glomerata*	*Polysiphonia fucoides*	Industrial (mechanical) collection of biomass	
	Extraction Soxhlet method, solvent: EtOH				SC-CO_2_
Chlorophyll *a*	0.28 ± 0.02	0.30 ± 0.01	0.22 ± 0.01	0.24 ± 0.02	0.32 ± 0.01
Carotenoids	0.07 ± 0.01	0.08 ± 0.01	0.13 ± 0.01	0.06 ± 0.01	0.09 ± 0.01

### Total phenolics and flavonoid content; antioxidant properties of marine and freshwater algae extracts

The applied colorimetric method allowed the determination of total polyphenolic compounds and flavonoids in the tested samples (Table 6). The concentrations of these substances ranged from 20.32–41.73 mg GAE g^−1^ for polyphenols and 1.08–1.72 mg QE g^−1^ for flavonoids in the samples of extracts. The SFE-CO_2_ extract, which was analyzed after 1 year showed the same amount of polyphenolic compounds (Michalak et al. 2016). The highest content of polyphenols was extracted from the sample of Baltic algae using UAE and the smallest from the sample of the same biomass, but prepared with SFE-CO_2_.

**Table 6:** Comparison of total phenolics and flavonoids content and antioxidant activity of macroalgal extracts. Values presented are mean ± standard deviation, *n* = 3

Type of extract	Extract of mix of Baltic algae		Extract of freshwater *Cladophora glomerata*	
Compound content/activity	SFE-CO_2_	UAE	SFE-CO_2_	UAE
Total phenolics [mg GAE g^−1^]	20.32 ± 0.63	41.73 ± 1.80	25.22 ± 1.11	21.50 ± 1.71
Total flavonoids [mg QE g^−1^]	1.51 ± 0.03	1.12 ± 0.08	1.08 ± 0.04	1.77 ± 0.10
Activity towards DPPH for 200 μL of sample [%]	58.72 ± 2.79	72.90 ± 1.36	66.47 ± 2.34	65.23 ± 2.58
Concentration of antioxidant—C [mg TEAC (100 mL)^−1^]	5.89 ± 0.36	7.72 ± 0.18	6.89 ± 0.30	6.73 ± 0.33

*GAE* gallic acid equivalents, *QE* quercetin equivalents

All samples showed the antioxidant activity in the range of 59–73%. The highest activity was measured for the sample made out of Baltic algae mixture obtained by the UAE, approx. 73%, and the antioxidant concentration approx. 8 mg TEAC (100 mL)^−1^.

## Discussion

### Macroalgae characteristics

In August 2013, in the collected biomass from coastal sites in Sopot, the dominant algae species were filamentous algae from the genera *Cladophora* sp. and *Ulva* sp. with a large share of the red alga *Polysiphonia*. However, the filamentous algae disappeared in September when the number of *P. fucoides* thalli significantly increased. Irrespective of the collection period, the algae were rough to the touch, due to the presence of numerous epiphytes (diatom, green algae, and cyanobacteria) and macroalgal thalli incrusted on the surface of the cell walls (Michalak et al. 2016).

Filamentous forms of *C. glomerata* may be either attached to various substrates using rhizoids or loosely floating to form a mat. The color was usually dark green. It is a cosmopolitan species, common in marine and littoral ecosystems (estuaries), saline, and freshwater. It is common in the Polish Baltic Sea: the Gdańsk Gulf (Pliński and Jóźwiak 2004), east coast of the Pomorska Gulf (Rosińska et al. 2013), and Sopot (this study). It occurs particularly in highly morphologically transformed and eutrophic locations.

*Ulva intestinalis* is the most common *Ulva* species in Poland (Messyasz and Rybak 2009; Messyasz et al. 2015b) and often occurs with filamentous green algae, mainly *C. glomerata*. Its thalli are tubular and wrinkled, with numerous proliferations. Young thalli are attached to the substrate, whereas mature thalli float on the water surface. Its thallus is tapered at the scutellum, which attaches to the substrate and further expands and remains cylindrical up to the apical part. It is frequently found in the coastal zone of seas, oceans, and estuaries (Lee 1999; Kirchhoff and Pflugmacher 2002). *Ulva intestinalis* is a typical euryhaline species (Young et al. 1987). Therefore, its tolerance to salinity is high (Pliński and Hindák 2012). *Ulva intestinalis* grows best with in a salinity around 24‰, but it is also found in areas with lower values of salinity (Kim and Lee 1996). The alga is widespread, and in some places is growing massively on empty shells, larger stones, and port breakwaters. In the Polish part of the Baltic Sea, *U. intestinalis* was found in the littoral zone of water bodies in Władysławowo, Świnoujście, Kołobrzeg, Łeba, Mielno, Gdańsk Bay, and Puck Bay (Haroon et al. 1999; Pliński and Jóźwiak 2004; Pliński and Hindák 2012). In our samples, thalli of *U. intestinalis* were accompanied by *U*. *clathrata*, which has an entero-folding type of construction, and can be found in the submerged form and free-floating mats. It is a cosmopolitan euhalinity (salinity values above 28‰) species with a wide distribution in marine and brackish environments throughout the world (Haroon et al. 1999; Bäck et al. 2000; Pliński and Hindák 2012).

Red algae from the genus *Polysiphonia* occur in the entire Baltic Sea, and also on the Polish coast, (Pliński and Hindák 2012)*.* They form dense mats on a solid surface (e.g., stones), but also loosely floating mats, detached from the substrate. In favorable growth conditions, *P. fucoides* thalli are red and contain mostly the pigments (phycoerythrin and chlorophyll) that are necessary for photosynthesis. In contrast, in harsh environmental conditions, it produces and accumulates high amounts of xanthophyll and carotenoids, with a concomitant change in coloration of the cells and thalli from red to brown (Van den Hoek et al. 1995; South and Whittick 1996). In this research, the thalli of *P. fucoides* reached the length of 25 cm and were dark brown in color.

In freshwater Lake Oporzynskie, the presence of filamentous algae was visible in the form of compact and dense mats covering considerable surface area and occurring near the shore. The biomass of *C. glomerata* was common in conglomeration, which formed large surface mats that tightly covered the surface of the water of this lake.

All of these natural sources for obtaining the biomass of algae have their advantages and limitations. In the climatic conditions of Central Europe, easy acquisition of homogeneous species of algae allows obtaining the best quality material for industrial, agricultural, and cosmetology uses.

### Biologically active compounds in marine and freshwater algae extracts

In the supercritical extract of different species of seaweed, the natural bioactive compounds that could be potentially used for the food, pharmaceutical, and cosmetic industries were detected and characterized. In the present study, the extraction of fatty acids from algal biomass was performed by two methods: Soxhlet extraction with ethanol as a solvent and SFE with carbon dioxide as solvent. The algal biomass used for the extraction determines the composition of the extract and the ratio of concentration of unsaturated to saturated fatty acids, irrespective of the technique of extraction. The composition of the extract and concentration of saturated and unsaturated fatty acids depends on the type of algae biomass used for extraction, technique extraction, and solvent used for the extraction. The composition optimal for cosmetic industry had the extract obtained by supercritical CO_2_ extraction. We also found that the ratio of unsaturated to saturated fatty acids in this extract was about one, which means that the extracts obtained from marine monospecies or multi-species could be used in cosmetic formulations.

Investigation of biomass of freshwater macroalga *C. glomerata* as a source of fatty acids, amino acids, and other bioactives (Khalid et al. 2012; Messyasz et al. 2015a) has not been as common as that of marine algae (Elenkov et al. 1996; Pereira et al. 2012; Horincar et al. 2014). Harvesting of the freshwater algal biomass studied was made in 2013 and 2014. The above-described results indicate that, regardless of the period of growing, the quantitative composition of fatty acids in the extract from these algae obtained by both the classical Soxhlet apparatus and with the application of supercritical extraction was similar. Variable weather conditions and differences in water properties as a habitat during this period are crucial for the growth of algae in the wild and responsible in about 10% for the composition of the obtained extracts. The SC-CO_2_ extraction gives better results as compared to methods of extraction using Soxhlet apparatus, both in terms of the number of the extracted fatty acids and in terms of the concentration ratio of saturated to unsaturated fatty acids.

The extract obtained from algae biomass containing unsaturated fatty acids, especially omega-3, omega-6, and omega-9 are valuable material for the production of cosmetic products, as they are natural and environmentally friendly. Unsaturated fatty acids are highly beneficial for the skin, because they are part of its structure. The unsaturated fatty acids keep the skin firm and elastic and at the proper level of moisture and lubrication. The fatty acids slow down the aging process and help the skin cope with the adverse effects of the environment. A very important role in the skin and hair care has a mixture of exogenous unsaturated fatty acids. These acids are used topically and can be incorporated into cell membrane layers of corneocytes (Katsuta et al. 2005), thus preventing water loss and inhibiting the aging of the skin. For this reason, they are often referred to as a factor regulating the skin water and lipids. Creams, conditioners or masks containing a composition of active ingredients derived from algae biomass represents a new line of ecological cosmetics, which not only contribute to the improvement of our skin but also are sourced from renewable, natural biological materials.

### Antioxidant activity of marine and freshwater algae extracts

Several studies have been conducted in order to characterize and evaluate *Ulva* species. For instance two species of *U. clathrata* and *U. prolifera* were used from four different locations in Iran were subjected to the FC, TPC, and DPPH tests. Methanolic extracts were prepared and better results were observed for *U. clathrata*, 4.468 ± 0.379 mg GAE g^−1^, 45.577 ± 0.949 mg RE g^−1^ (rutin equivalent) and the radical scavenging activity with a low IC50 (the half-maximal inhibitory concentration) 0.715 ± 0.078 mg mL^−1^, respectively (Farasat et al. 2013). In another investigation, ethanolic extracts and fractions from solvents with various polarities from green seaweeds such as *E. compressa* (L.) Ness (syn. *Ulva compressa*), *Cladophera fulvescens*, *C. moniligera*, and *U. pertusa* were assessed for the same parameters. Samples from *U. pertusa* contained 9 mg GAE g^−1^ of phenolic compounds in ethanol, but this amount was approximately three times greater in the fraction from ethyl acetate and 27.4 mg QE g^−1^ of flavonoids in ethanol and 147 mg QE g^−1^ in ethyl acetate fraction. From among four tested species, all extracts and fractions from *U. pertusa* had the lowest content of polyphenols. The ethyl acetate fraction contained the highest amount of flavonoids in comparison to fractions in the same solvent from other species (Cho et al. 2010). Results of our study confirmed how strongly the extracted amounts of compounds depend on the species and type of solvent used for extraction. Interestingly, in the sample of Baltic algae investigated here, a higher content of polyphenols was obtained after UAE when compared to SFE-CO_2_. Differences between the results could be related to the instability of this group of compounds, age of the sample, the use of various extraction techniques, and the application of different materials. These groups of substances also show antioxidant activity; thus, the samples with high flavonoids and polyphenols content may exhibit strong radical scavenging activity in DPPH test or any other antioxidant protocol.

The levels of secondary metabolites from six species of edible seaweeds from different classes of algae were estimated. Total phenolic content in methanolic extracts of brown algae such as *Laminaria digitata*, *Laminaria saccharina*, *Himanthalia elongata*, red algae *Palmaria palmata*, *Chondrus crispus*, and the green macroalga *Ulva spirulina* ranged from 37.66 to 151.33 mg GAE g^−1^ of dry extract. The brown seaweed, *H. elongata*, exhibited the highest content of this group of substances and the lowest was found in the extract of *L. digitata.* Such a low level of polyphenols in *L. digitata* might be considered strange, because usually brown seaweeds contain more of these compounds in comparison to red and green seaweeds. However, two other brown seaweeds from this research had significantly higher content of polyphenols. Total flavonoids in the seaweeds ranged from 7.66 to 42.5 mg QE g^−1^ of extract. Again, the highest value was observed for *H. elongata.* For the only green seaweed in this study, this value was 19.05 ± 0.73 mg QE g^−1^ of extract (Cox et al. 2010). This value is more than tenfold greater than the content of flavonoids in the methanolic extract of *Cladophora* spp. (1.77 ± 0.10 mg QE g^−1^) from this research.

Ethanolic extracts from *C*. *glomerata* collected in Iran contained significantly higher amounts of phenolic compounds and flavonoids, 3077 ± 105 mg GAE g^−1^ and 595 ± 23 mg QE g^−1^, respectively, than both extracts we studied (Soltani et al. 2011).

The content of biologically active substances from natural origins depends on the species, site of the material collection, and type and conditions of extraction (temperature, solvent, time). Many authors who worked on the assessment of seaweeds as the potential sources of valuable compounds have used the colorimetric methods, mainly describing the concentration of phenolic compounds and flavonoids in combination with radical scavenging activity tests (Soltani et al. 2011; Tabarsa et al. 2012; Vijayabaskar and Shiyamala 2012; Chakraborty et al. 2013; Mezghani et al. 2013; Gouda et al. 2014; Heffernan et al. 2014; Chakraborty et al. 2015; Farah Diyana et al. 2015; Gall et al. 2015; Machu et al. 2015; Mannino et al. 2015). In some cases, screening tests have been performed for extracts in different solvents, which gives preliminary information about the content of various classes of compounds in seaweed (Jeeva et al. 2012; Domettila et al. 2013; Whankatte and Ambhore 2016). Our investigation has demonstrated the antioxidant activity on a level from 59 to 73% with the highest value (approx. 8 mg TEAC (100 mL)^−1^) for the sample of Baltic algae mixture. This may be due to the presence of polyphenols in the extract, which increase the antioxidant capacity, but can be also related to the presence of metal ions (Michalak and Chojnacka 2016). Higher activity towards DPPH may be caused by the diversity of species and the preferable polarity of the extrahent-methanol, which increases the content of extracted polyphenols.

On the basis of our results, a strong correlation between the antioxidant activity of the sample and the content of phenolic compounds can be confirmed. However, it should be taken into consideration that the observed free radical scavenging activity may have resulted also from the presence of other compounds in the extract having antioxidant nature. Therefore, the calculated total content of antioxidants, expressed as Trolox equivalents, gives information about all antioxidants in the sample. Tests with DPPH, as other methods used to assess the antioxidant properties, are sensitive to chemical disturbances; thus, the results of measurement of complex matrices in our extracts may be flawed. In addition, a limitation of this method is the fact that DPPH dissolves only in organic solvents, which does not allow determination of hydrophilic antioxidants (Cybul and Nowak 2008). This is another reason why the results obtained by the Folin-Ciocalteu method and DPPH test are independent of each other and why the determined antioxidant capacity can be attributed not only to the phenolic compounds.

In conclusion, a great number of studies concerning the evaluation of seaweed for the content of biologically active compounds have proved that these organisms have a very high potential for their applications in many branches of industry, medicine, and human well-being. We have shown here that the composition of the extract and concentration of saturated and unsaturated fatty acids depends on the type of algae biomass used for extraction, extraction technique, and solvent used for the extraction. Moreover, the optimal composition for cosmetic uses was found in the extract obtained from supercritical CO_2_ extraction. The content of polyphenols in the extracts, which are the active ingredients showing antioxidant activity, ranged from approximately 2 to 4%. Flavonoids represent less than 10% of the total content of polyphenolic compounds, which is consistent with the literature data concerning the proportions of these compounds in seaweed. Regardless of the method used, the extraction efficiency of polyphenols from freshwater *C. glomerata* was on a similar level, whereas for the mixture of Baltic algae, the efficiency was higher for the extracts obtained by UAE. This observation may be explained by the fact, that the mixture contains different species of Baltic algae, so the composition of extracted biomass is more diverse. Another explanation is that the use of the polar solvent in UAE is a better extraction medium for polyphenols when compared to CO_2_.
